# Quasi-steady state aerodynamics of the cheetah tail

**DOI:** 10.1242/bio.018457

**Published:** 2016-07-13

**Authors:** Amir Patel, Edward Boje, Callen Fisher, Leeann Louis, Emily Lane

**Affiliations:** 1 Department of Electrical Engineering, University of Cape Town, Cape Town 7701, South Africa; 2 Department of Integrative Biology, University of California, Berkeley, Berkeley, CA 94720-3140, U.S.A; 3 Department of Epidemiology, National Zoological Gardens of South Africa, Pretoria 0001, South Africa

**Keywords:** Cheetah, Tail, Aerodynamics, Inertia, Manoeuvrability

## Abstract

During high-speed pursuit of prey, the cheetah (*Acinonyx jubatus*) has been observed to swing its tail while manoeuvring (e.g. turning or braking) but the effect of these complex motions is not well understood. This study demonstrates the potential of the cheetah's long, furry tail to impart torques and forces on the body as a result of aerodynamic effects, in addition to the well-known inertial effects. The first-order aerodynamic forces on the tail are quantified through wind tunnel testing and it is observed that the fur nearly doubles the effective frontal area of the tail without much mass penalty. Simple dynamic models provide insight into manoeuvrability via simulation of pitch, roll and yaw tail motion primitives. The inertial and quasi-steady state aerodynamic effects of tail actuation are quantified and compared by calculating the angular impulse imparted onto the cheetah's body and its shown aerodynamic effects contribute to the tail's angular impulse, especially at the highest forward velocities.

## Abstract (precis)

**Summary:** The aerodynamics of the cheetah tail are non-trivial. This paper is the first to quantify the effect of aerodynamic forces from a cheetah's tail during rapid manoeuvres.

## INTRODUCTION

**Fig. 1.**
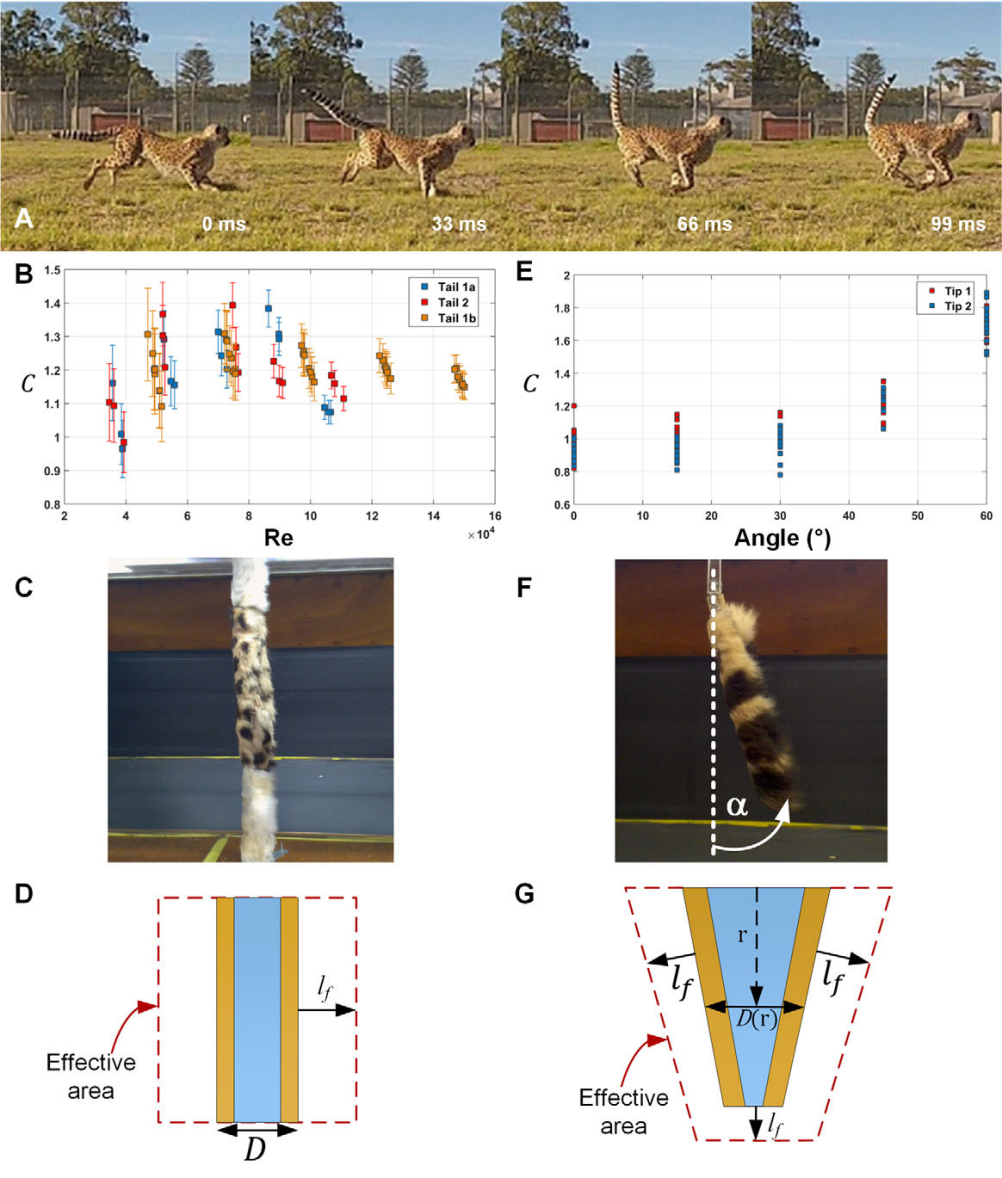
**Example of cheetah tail motion with aerodynamic data and methodology shown.** (A) Montage of a captive cheetah chasing a lure illustrating the motion of the tail in the sagittal plane. (Video for this is provided as Movie 1). (B) Drag coefficient of a furry cylinder showing the effects as a function of Reynolds number. Results for the larger diameter tail, labelled ‘Tail 1b’. Error bars (1*σ*) are shown for each measurement. (C) The furry cylinder test rig with central furry test-piece (spotted) free to rotate about a bearing is depicted. Faux-fur cylinders on each end are fixed to the wind tunnel frame to eliminate end effects. (D) The furry cylinder drag normalised to produce an effective area which resulted in an effective fur length (*ℓ_f_*) for each tail. (E) Aerodynamic coefficients of morphometric tips over 0° to 60° angle of inclination. (F) The morphometric tail rig used to measure aerodynamic coefficient at varying angles of inclination and airspeed. (G) The tail aerodynamic force was normalised to produce an effective area of a truncated cone which resulted in an effective fur length (*ℓ_f_*) for each tail tip.

The cheetah's hunting success has been attributed to both its speed and agility during acceleration and turning, with tail movements often being associated with these manoeuvres (Wilson, et al., 2013). During pursuit of prey the tail is swung from one side of the body to the other (in the roll axis) for turn initiation at high-speed (P. Hudson, PhD Thesis, Royal Veterinary College, London, 2011) and upwards or downwards (in the sagittal plane) during rapid acceleration and braking manoeuvres (Fig. 1A; Movie 1). These movements have the potential to exert inertial forces and torques on the body (Wilson et al., 2013) similar to those observed in lizard tails (Libby et al., 2012). The cheetah tail is commonly referred to as functioning as a *rudder* (used to change the heading of body) or a *counterweight* (used for balance) (Eaton, 1972; Thompson, 1998). Actuated tails on cheetah-inspired robots provide inertial compensation, stabilising the platforms when making aggressive manoeuvres (Patel and Braae, 2013, 2014). Measurements of cheetah tails however, indicate that the tail is relatively light, and, as most of its mass is located near the base (P. Hudson, PhD Thesis, Royal Veterinary College, London, 2011), the relevant moment of inertia is modest. Aerodynamic effects of the cheetah tail during rapid manoeuvres have not been investigated and our hypothesis is that the long, furry tail generates aerodynamic forces that contribute to the angular impulse (especially at high speeds), thereby assisting manoeuvrability. In an aerodynamic study of tarsiers, effects of tails were found to be negligible because of low airspeed (Peters, 1985) but in cockroach-inspired robots with sail-like tails they were significant and allowed rapid turns (Kohut et al., 2013).

In this paper, we report on measurements of the aerodynamic force of two distinct sections of the tail (mid-section and tip) in a series of wind tunnel tests. Using this data, we ran dynamic simulations of a rigid tail executing swinging motions to estimate the contribution of aerodynamic forces on the body and draw conclusions from the results.

## RESULTS AND DISCUSSION

### Straight section measurements

The effective area was determined for each tail so as to minimise the weighted (via propagation of the wind speed and force measurement accuracies as independent statistical quantities) mean square error in Eqn (1) with fixed *C*=1.2. After accounting for the skin thickness, the effective fur lengths were *ℓ_f_*=8.8 mm and *ℓ_f_*=11.3 mm for Tails 1 and 2, respectively. These lengths are less than the lengths of the guard hairs (ranging from 22 mm to 39 mm over both tails; see Fig. S1). The effective area in the mid-section of both tails is nearly double (1.8 times) the frontal area of the tail without fur, allowing an increase in aerodynamic forces without significant mass penalty. The final drag coefficient data with effective areas determined above are depicted in Fig. 1B.

### Tail tip measurements

The cheetah tail tip effective areas (calculated as above) resulted in fur lengths of 11.9 mm and 8 mm for Tails 1 and 2, respectively and the aerodynamic coefficient data is depicted in Fig. 1E. Again, these fur lengths are plausible based on the measured guard fur (ranging from 31 mm to 50 mm). During the wind tunnel tests, we observed that the very long fur on the tail tip tended to collapse at higher airspeeds (>20 m/s) but this deformation of the tip fur was not observed in video footage of cheetahs performing rapid manoeuvres. This implies that the tail may potentially exert even greater forces on the body than we have estimated.

### Simulation

**Fig. 2.**
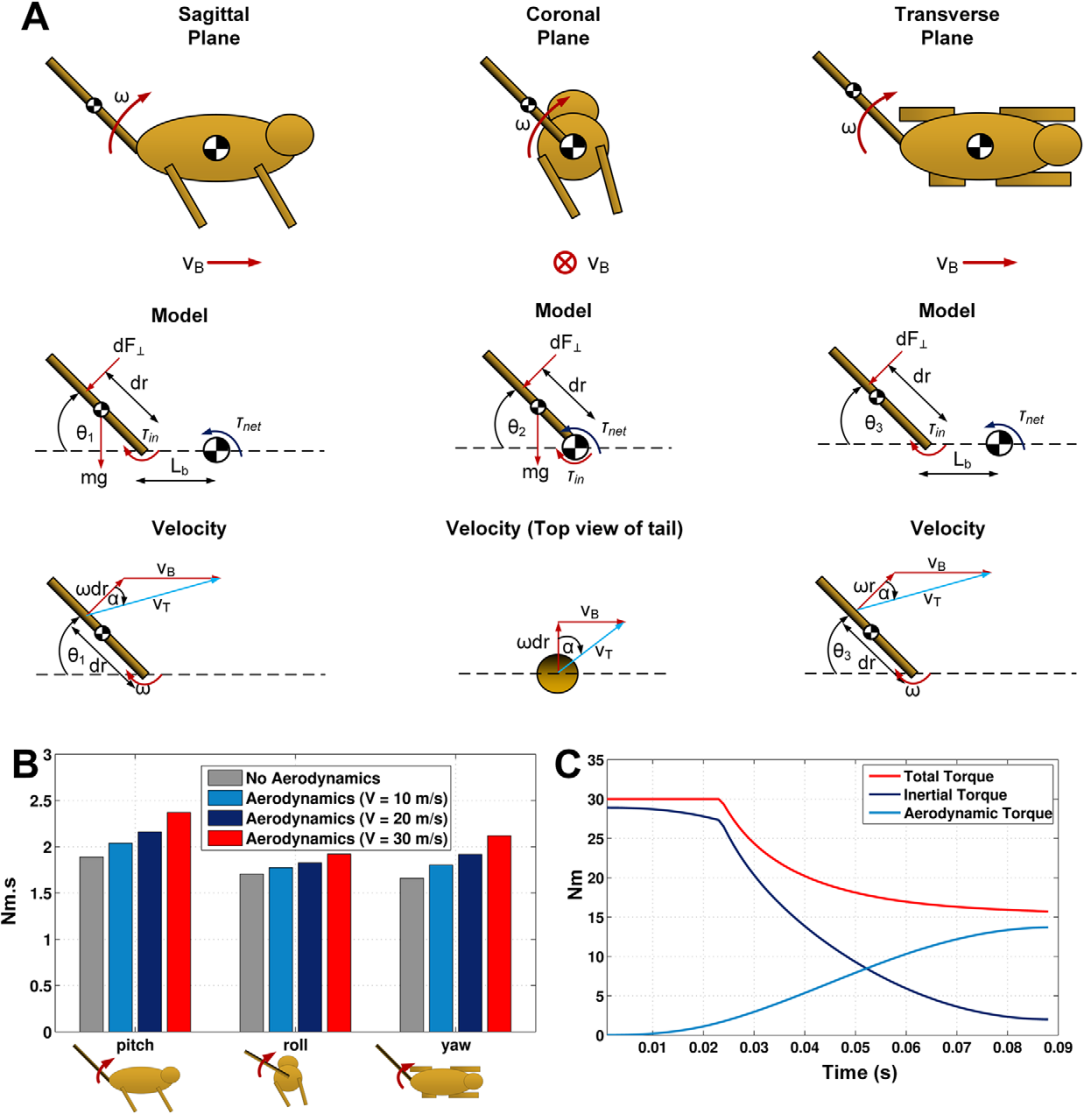
**Dynamic models and simulation results.** (A) Three planar models for each of the tail motions (pitch, roll and yaw) are shown. Each model has its own distinct forces as well as velocity of the tail. (B) The angular impulse during various tail manoeuvres shows that the aerodynamic effects notably assist the motions when the body is moving forward at a high velocity. (C) An example simulation of the torque imparted on the body by swinging the tail in the roll axis at 30 m/s forward velocity.

The simulation results are summarised in Fig. 2B. As can be seen, aerodynamic effects make a contribution to the angular impulse, in the pitch, roll and yaw cases (respectively 26%, 12.6% and 28% of the total 30 m/s). Initially the effect of the tail is inertial, but as the inertial effects decrease, the total torque is maintained by an increase in the aerodynamic torque (Fig. 2C). Tail motion in the pitch axis has been attributed to both increasing pitch stability and lowering the peak forces on the front legs (Wilson et al., 2013). In addition, by yawing the tail in the aerial phase the cheetah can redirect its body heading and by rolling the tail it can bank the body appropriately into a turn (P. Hudson, PhD Thesis, Royal Veterinary College, London, 2011) or counteract the destabilising effect of the centrifugal force (Patel and Braae, 2013). In all cases, the aerodynamic force also has a braking effect.

We have not considered non-linear, time varying and distributed aerodynamic effects. Nevertheless, our first order, quasi-steady state results clearly support the hypothesis that aerodynamic effects of the cheetah's long, furry tail contribute to the angular impulse that can be applied to the body, especially at higher speeds. Both inertial and aerodynamic effects must therefore be considered in modelling the use of the cheetah tail for manoeuvring tasks. Our results further support the observations that the cheetah tail can be used as a ‘rudder’ to contribute to fast change of heading, and as a ‘stabiliser’ during rapid acceleration and turning.

## METHODS

### Animal measurements

The use of the cheetah tails complied with the University of Cape Town Science Faculty Ethics policy. Tails from two euthanized cheetahs were obtained from the National Zoological Gardens of South Africa. The cheetah tails were removed from the intervertebral disc between S3 and C1, weighed (Boeco BBL61, 0.1 g) and measured. The vertebrae of Tail 1 were individually measured to calculate the moment of inertia about the base (via Steiner's theorem). Our measurements are compared to previous results in Table 1.

**Table 1.**
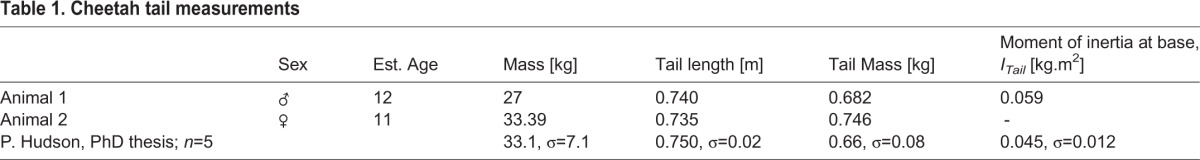
**Cheetah tail measurements**

### Wind tunnel tests

The aerodynamic force normal to a section of the tail is the result of movement of the tail relative to the air (Anderson, 2010). As a first approximation, this force scales with the square of the tail's airspeed (the magnitude of the vector sum of the longitudinal, rotational and wind velocities) and is approximated by:
(1)
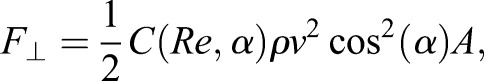
where *ρ* is the fluid (i.e. air) density; *ν* is the relative fluid speed; *α* is the angle of inclination, *C*(*Re*,*α*) is the coefficient of aerodynamic force and is dependent on the Reynolds number, *Re*=*vρD*/*μ* (where, *D* is the characteristic length and *μ* is the dynamic viscosity), that normalizes scale effects.

As the cheetah tail does not have a distinct fur-air boundary, we define the notion of an effective area, *A_eff_*, so that the aerodynamic force is the same as that of a smooth cylinder of the same area,
(2)
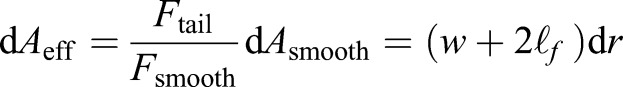
where, *dr* is the incremental length along the tail, *w* is the diameter of the tail at the skin-fur boundary (excluding the fur), and *ℓ_f_* is the effective length of the fur. For the Reynolds number we use *D*=*w*+2*ℓ_f_* as the characteristic length. This concept is depicted in Fig. 1D. The wind tunnel used is a closed circuit, recirculating type with throat dimensions 590×760 mm giving a blockage factor of ∼10%. Each force measurement was obtained from a purpose-built load cell.

We measured the drag of a section of a furry cylinder normal to the airflow, for varying wind speeds up to 30 m/s. This is comparable to the average hunting speed of ∼20 m/s (Wilson et al., 2013) executing a tail flick of ∼17 rad/s at a length of 0.7 m (P. Hudson, PhD Thesis, Royal Veterinary College, London, 2011). A 300-mm long piece of 32-mm diameter PVC pipe (from the average diameter of Tail 1 of ∼31 mm, Table S2), was covered with cheetah tail pelt from the middle section of each tail. To minimise end effects, dummy fur-covered cylinders were fixed above and below the test section as illustrated in Fig. 1C. The fur was compressed using Vernier callipers to estimate skin-fur boundary diameter. A third set of measurements was made with pelt from Tail 1 but using a 50-mm diameter pipe, giving a higher Reynolds number at the same airspeed.

The tip of the tail is bushy, typically experiences the largest speeds, has the largest lever arm, and complicated span-wise or end-effects. Based on the approximate geometry of the last ten caudal vertebrae of Tail 1 (Tables S1 and S2), a truncated cone (Fig. 1G) of length 238 mm with initial diameter 18 mm and tip diameter 5 mm was constructed from wood and covered with pelt. The tail tip test measured the force normal to the tail tip at varying angles of inclination (0° to 60°) and wind speeds (up to 30 m/s) (Fig. 1F). We assumed that the tip is extended by the fur length (Fig. 1G), and neglected span-wise effects of the tapered cone and tip end. Although these are approximations for a very complex underlying problem, the morphometric tail tips have comparable geometry and scale to actual tails and we therefore expect the force data to be recovered in calculations for other tail tips.

### Dynamic models

The motion of any animal in its natural environment is very complex. From observations of captive cheetahs at the Cheetah Outreach (Somerset West, South Africa) and from wildlife documentaries (Table S3), we abstracted the following simplified motion primitives to aid understanding; pitching in the sagittal plane, yawing in coronal plane, and rolling in the transverse plane. We developed rigid tail models using Euler–Lagrange dynamics for each of the three motions (see Fig. 2A). The assumption that the tail is rigid is a reasonable starting point as it appears that the tail is often held rigidly when swinging (P. Hudson, PhD Thesis, Royal Veterinary College, London, 2011). For the pitch and yaw case it is assumed that the tail is attached at some distance, *L_b_*, from the centre of mass (CoM) of the body. For each of the models, the velocity of the tail is calculated using geometry.

To quantify the reaction torque on the body, a redundant body angle coordinate was introduced and constrained to be zero. For each model, the generalized coordinate vector is
(3)
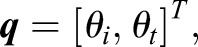
where *θ_i_* is the body angle in the relevant axis (pitch, roll or yaw respectively) and *θ_t_* is the tail angle for the particular axis motion.

The aerodynamic force (*F*_⊥_) and subsequent torque (*T_A_*) is then included as
(4)
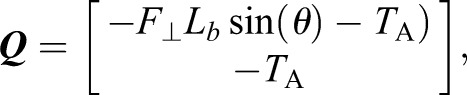
where *L_b_* is zero for the roll (coronal plane) model. At each simulation step, the tail was discretised and aerodynamic behaviour found by numerical integration along its length (*L_t_*) using the effective area and coefficient (C≈1.2 for a smooth cylinder; Anderson, 2010) to obtain the aerodynamic force and torque as follows:
(5)
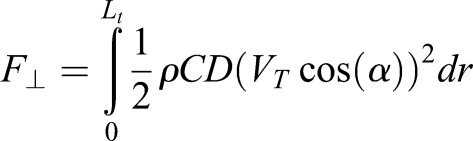

(6)
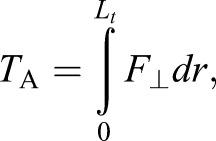
where *V_T_* is the total velocity magnitude and it is assumed that the tail is a constant cylinder. Cheetah specific muscle power has been reported as 600 W/kg (West et al., 2013) and the tail is actuated by approximately 680 g of muscle (P. Hudson, PhD Thesis, Royal Veterinary College, London, 2011). We have used *P_max_*=400 W for the power limit in our simulations. The torque limit however is unknown but to give plausible simulations, we used *T_max_*=30 Nm, which corresponds to swings covering 90° in 0.07 s (including power limits at higher speeds) observed in wildlife documentary video footage (Table S3). This average rate (22 rad/s) is supported by 17±4 rad/s average speed reported by P. Hudson (PhD Thesis, Royal Veterinary College, London, 2011) for 180° swings. The input torque command was limited as follows:
(7)
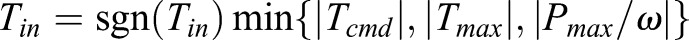
The tail was commanded with the maximum input torque, *T_cmd_*=30 Nm, and the simulation terminated when the tail angle reached 90°. To quantify the effects of pitching, rolling and yawing motions, each model was simulated (using Tail 1 data) in Matlab (ode45 variable step solver) for varying body velocities (*V_B_*), with and without aerodynamic force. To compare the results, the angular impulse was calculated as,
(8)
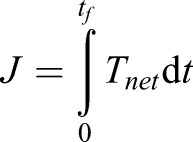
where *t_f_* is the simulation time and *T_net_* is the reaction torque on the CoM in each respective axis.
